# Precise prediction of cerebrospinal fluid amyloid beta protein for early Alzheimer's disease detection using multimodal data

**DOI:** 10.1002/mco2.532

**Published:** 2024-04-19

**Authors:** Jingnan Sun, Zengmai Xie, Yike Sun, Anruo Shen, Renren Li, Xiao Yuan, Bai Lu, Yunxia Li

**Affiliations:** ^1^ Department of Biomedical Engineering Tsinghua University Beijing China; ^2^ Department of Neurology, Shanghai Pudong Hospital Fudan University Pudong Medical Center Shanghai China; ^3^ Shanghai Key Laboratory of Vascular Lesions Regulation and Remodeling Shanghai China; ^4^ School of Pharmaceutical Sciences Tsinghua University Beijing China; ^5^ Beijing Academy of Artificial Intelligence Beijing China; ^6^ Department of Neurology Tongji Hospital Tongji University Shanghai China

**Keywords:** amyloid beta protein, cerebrospinal fluid, EEG, MRI

## Abstract

Alzheimer's disease (AD) constitutes a neurodegenerative disorder marked by a progressive decline in cognitive function and memory capacity. The accurate diagnosis of this condition predominantly relies on cerebrospinal fluid (CSF) markers, notwithstanding the associated burdens of pain and substantial financial costs endured by patients. This study encompasses subjects exhibiting varying degrees of cognitive impairment, encompassing individuals with subjective cognitive decline, mild cognitive impairment, and dementia, constituting a total sample size of 82 participants. The primary objective of this investigation is to explore the relationships among brain atrophy measurements derived from magnetic resonance imaging, atypical electroencephalography (EEG) patterns, behavioral assessment scales, and amyloid β‐protein (Aβ) indicators. The findings of this research reveal that individuals displaying reduced Aβ1‐42/Aβ‐40 levels exhibit significant atrophy in the frontotemporal lobe, alongside irregularities in various parameters related to EEG frequency characteristics, signal complexity, inter‐regional information exchange, and microstates. The study additionally endeavors to estimate Aβ1‐42/Aβ‐40 content through the application of a random forest algorithm, amalgamating structural data, electrophysiological features, and clinical scales, achieving a remarkable predictive precision of 91.6%. In summary, this study proposes a cost‐effective methodology for acquiring CSF markers, thereby offering a valuable tool for the early detection of AD.

## Graphical abstract

The diagnosis of Alzheimer's disease (AD) typically relies on costly and invasive cerebrospinal fluid (CSF) markers, causing discomfort to patients. Sun et al. propose a cost‐effective method for early detection that utilizes a random forest approach to accurately estimate the Aβ1‐42/Aβ‐40 content. The method is based on noninvasive techniques including EEG and MRI and was tested on a sample of 82 subjects, providing valuable diagnostic information.

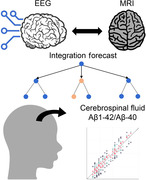

## 1 INTRODUCTION

Alzheimer's disease (AD) is a severe and debilitating neurodegenerative disorder that manifests as a gradual decline in cognitive function and memory loss.^1^ It is a leading cause of disability and mortality worldwide and is expected to increase in prevalence in the coming years, posing significant challenges for individuals, families, and healthcare systems.^2^ The pathophysiology of AD involves the accumulation of amyloid beta (Aβ) protein and tau protein in the brain,^3^ leading to the formation of plaques and tangles, respectively.^4^ This process results in the progressive degeneration of neurons, leading to cognitive impairment and dementia.^5^ The exact causes of AD are not fully understood, but genetic, environmental, and lifestyle factors are thought to play a role.^6^, ^7^ Despite significant advances in our understanding of AD, there is currently no cure for the disease. Treatment options include medication, cognitive and behavioral therapies, and lifestyle modifications, but these interventions are mainly palliative and aimed at managing symptoms rather than reversing or halting the disease's progression. Therefore, early detection and diagnosis of AD are crucial for effective management and treatment of the disease.^8^

The diagnosis of AD is primarily dependent on cerebrospinal fluid (CSF) indicators, positron emission tomography images, and magnetic resonance imaging (MRI) structural images, as per the National Institute on Aging‐Alzheimer´s Association (NIA‐AA) Framework.^9^ Among these, CSF biomarkers, such as Aβ1‐42, Aβ1‐40, and total tau (t‐tau), are the most specific and clinically significant for the diagnosis of AD. Although Aβ is a metabolite of the neural system, its massive deposition can be neurotoxic and affect the normal activity of glial cells and neurons. Recent studies have emphasized the role of CSF biomarkers, particularly Aβ42 and tau, in the diagnosis and progression prediction of AD. These biomarkers have been extensively validated and are considered key indicators in the clinical context of AD.^10^ Decreased values of Aβ1‐42/Aβ1‐40 in the CSF imply the deposition and damage of Aβ1‐42 in the neural system.^11^ Therefore, Aβ1‐42/Aβ1‐40 is a sensitive biomarker that can characterize the disease process of AD. Additionally, the discovery of novel CSF biomarkers using machine learning techniques has shown promise in predicting the rate of cognitive decline within dementia patients.^12^, ^13^

However, the collection of CSF is invasive and painful, and the testing can be expensive. As Aβ1‐42/Aβ1‐40 is one of the criteria for the recognition of AD, it would be of great clinical importance to develop a more convenient and patient‐friendly method to obtain this metric, which will contribute to early detection of the disease. MRI is an advanced neuroimaging technique used in the diagnosis of AD.^14^, ^15^ This technique measures the spontaneous low‐frequency fluctuations in the blood oxygen level‐dependent signal in the brain.^16^ Studies have shown that MRI is useful in detecting changes in the default mode network (DMN),^17^ functional connectivity,^18^ and ventricular structure,^19^ which may be associated with specific AD symptoms, such as executive dysfunction and memory impairment. Although MRI can detect clinically relevant indicators in the diagnostic framework, such as ventricular atrophy, it cannot be used as a standalone diagnostic tool because the phenomena mentioned above may not be sufficiently specific to AD.

The electroencephalogram (EEG) is a widely used method for obtaining physiological signals from the brain.^20^ It has gained popularity in neural engineering^21^, ^22^ and clinical research^23^ due to its noninvasive nature, ease of application, and high temporal resolution. EEG can detect subtle changes in the nervous system, information transmission, network evolution, and other system properties. Given the multitude of studies that have investigated the role of EEG in early monitoring, treatment intervention, development evaluation, and drug research and development for AD, it is expected that EEG will be included in the AD diagnostic system.^24^, ^25^, ^26^, ^27^ However, the spatial resolution of EEG is not ideal despite its significant advantage in the temporal dimension.

The ratio of Aβ1‐42/Aβ1‐40 in CSF serves as a specific marker for AD and exhibits a strong correlation with the extent of amyloid deposition in the cortex and ventricles.^28^, ^29^ This aggregation of proteins within the brain reflects the systemic damage, making it a valuable means to assess AD progression. The objective of this study was to predict Aβ1‐42/Aβ1‐40 levels in CSF using two cost‐effective and noninvasive techniques, MRI and EEG. This approach eliminates the potential hazards and expenses associated with surgical CSF collection. The spatial resolution provided by MRI compensates for the spatial information lacking in EEG, while the high temporal resolution of EEG complements the data obtained from MRI. Initially, we analyzed several features associated with Aβ1‐42/Aβ1‐40 indexes in EEG and MRI, and subsequently predicted the Aβ1‐42/Aβ1‐40 indexes using a feature engineering approach. Our approach offers a noninvasive, convenient, and more accessible alternative. By utilizing EEG and MRI, patients who perceive initial cognitive difficulties can undergo these tests as a preliminary screening method. The results from these tests can predict CSF biomarker levels, which are crucial in the diagnosis of AD. Based on these predictions, patients and healthcare providers can make informed decisions about whether to proceed with more detailed and invasive diagnostic procedures. This strategy not only reduces the need for early‐stage patients to undergo invasive testing but also provides a pathway for earlier intervention. By identifying potential risks at a stage where patients are only beginning to experience cognitive changes, there's a greater chance of implementing effective treatment and management strategies that could slow the progression of the disease.

## 2 RESULTS

### 2.1 Variation of EEG energy and complexity

In our study sample of 82 individuals, the mean age was 70.57 years, with a slight female majority (54.9%). Body mass index averaged at 23.15, and participants had a median education of 12 years, with most having at least a high school diploma. Lifestyle factors indicated low tobacco and alcohol use, with the majority being nonusers as shown in Figure 5. Medical history data showed that most participants had no history of stroke, hypertension, diabetes, or dyslipidemia. Detailed participant characteristics, clinical information, and full neuroimaging data are available in the Table S1.

**FIGURE 1:**
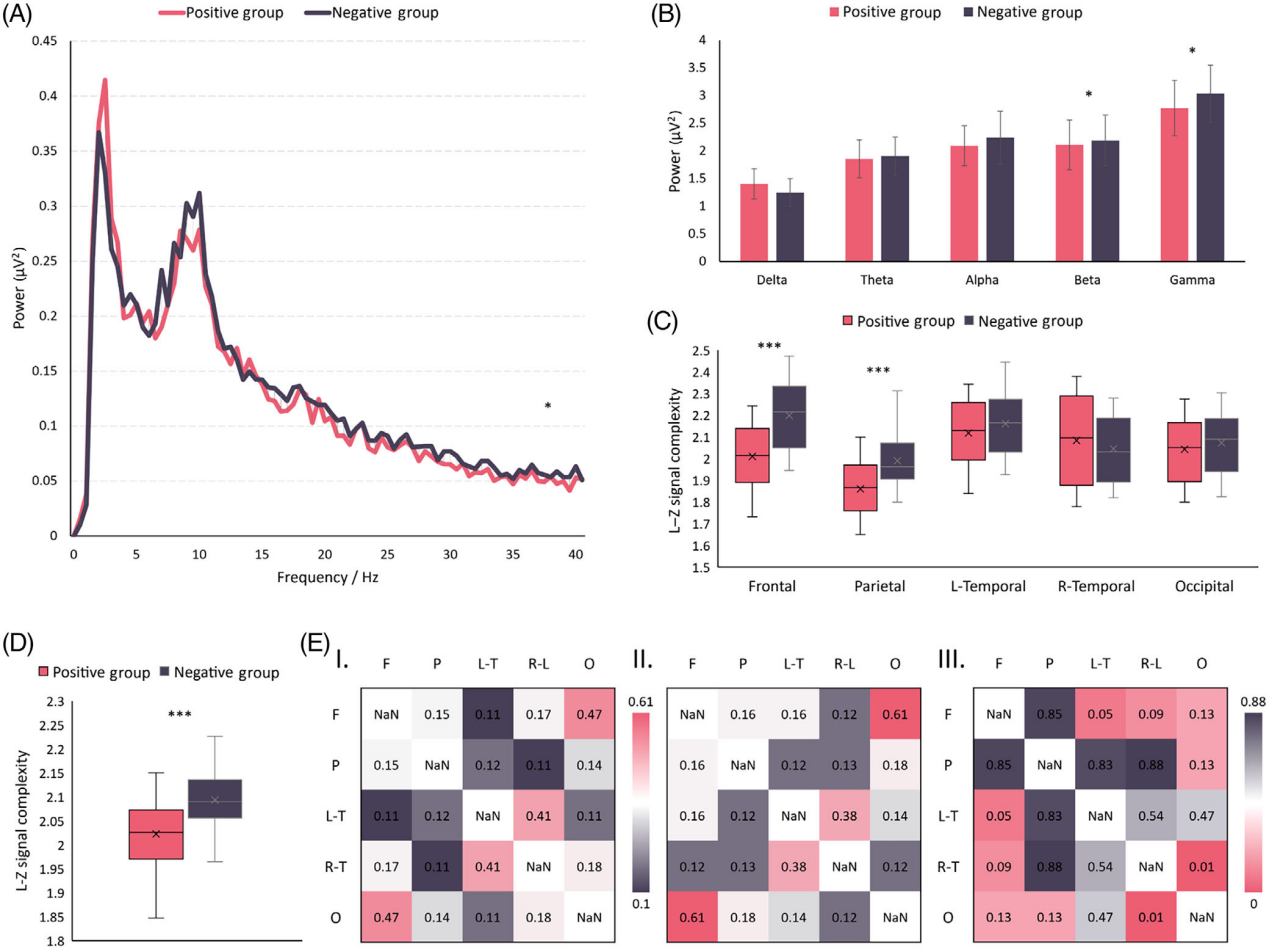
Results of EEG energy and complexity analysis. (A) Spectrograms are presented for the positive and negative groups. The energy–frequency curve for the positive group is shown in magenta, while the energy–frequency curve for the negative group is displayed in black. Notably, there exists a significant disparity in the distribution of the curves between the two groups. (B) Bar graphs are utilized to depict the total energy of the positive and negative groups at each frequency band. The magenta bar represents the positive group, whereas the black bar represents the negative group. In comparison with the negative group, the positive group demonstrates a noteworthy reduction in total energy within the beta and gamma frequency bands. (C) Box plots illustrate the L–Z signal complexity in different brain regions for both the positive and negative groups. Specifically, the positive group exhibits a considerable decrease in signal complexity within the prefrontal region. (D) Box plots portray the overall L–Z signal complexity in the positive group versus the negative group. The positive group displays a decrease in the mean and an increase in the variance of signal complexity when compared with the negative group. (EW) Mean mutual information matrices (I and II) are presented for the brain regions in the positive and negative groups, respectively, while matrix III depicts the *p* values obtained through a *t*‐test conducted between the two mutual information matrices. F stands for frontal, P for parietal, L‐T for left‐temporal, R‐T for right‐temporal, and O for occipital. The above summary results include a total of 82 participants (*n* = 82).

To investigate the association between the Aβ1‐42/Aβ1‐40 index and brain activity, we conducted an EEG recording analysis on our subjects. The subjects were divided into two groups based on their Aβ1‐42/Aβ1‐40 index values: subjects with Aβ1‐42/Aβ1‐40 ≤ 0.1 constituted the positive group, comprising a total of 47 subjects, while those with Aβ1‐42/Aβ1‐40 > 0.1 formed the negative group, totaling 35 subjects. Figure 1 illustrates the energy analysis conducted on these two groups, with Figure 1(A) displaying the spectrum of the groups. Statistical testing, specifically a paired t‐test, was performed on the energy distribution curves, and the results indicated a significant difference (*p* = 0.0103 < 0.05). Furthermore, we calculated the mean total energy within different frequency bands for both groups, as depicted in Figure 1(B). The frequency bands analyzed included delta (0.5–3 Hz), theta (3–8 Hz), alpha (8–12 Hz), beta (12–20 Hz), and gamma (20–40 Hz). No statistically significant differences were observed in the delta, theta, and alpha bands. However, the positive group displayed significantly lower mean total energy values than the negative group in the higher frequency ranges. Specifically, within the beta band, the positive group exhibited mean total energy of 2.11 µV^2^, which was significantly lower than the negative group's mean total energy of 2.19 µV2 (*p* = 0.02 < 0.05). In the gamma band, the mean total energy of the positive group was 2.77 µV^2^, significantly lower than the negative group's mean total energy of 3.03 µV2 (*p* = 0.04 < 0.05). Additionally, the positive group demonstrated lower total energy (10.23 µV^2^) than the negative group (10.61 µV^2^) across the entire 0–40 Hz frequency range. These findings indicate a gradual decrease in EEG total energy with declining Aβ1‐42/Aβ1‐40 values, predominantly evident in the higher frequency bands such as beta and gamma.

To examine the changes in brain activity, we conducted an analysis of the Lempel–Ziv (L–Z) signal complexity of EEG and evaluated the mutual information. Figure 1(C) presents the box plots illustrating the L–Z signal complexity for each lead within the five distinct brain regions. Additionally, Figure 1(D) displays the box plots demonstrating the overall L–Z complexity for each lead. The mean complexity values of the positive group exhibited varying degrees of decrease across all five brain regions in comparison with the negative group. However, a statistically significant decrease was observed in the frontal lobe (*p* = 0.000005 < 0.001) and the parietal lobe (*p* = 0.000007 < 0.001). This finding suggests that the complex cognitive processes in the frontal and the parietal regions were suppressed. Moreover, Figure 1(E) I and II illustrate the mutual information matrices between each brain region for the positive and negative groups, respectively. Furthermore, Figure 1(E) III presents the *p*‐value matrix obtained by performing *t*‐tests on the two mutual information matrices. The results reveal significantly lower mutual information values between the frontal and left temporal regions, as well as between the occipital and right temporal regions. The transmission of neural information between the frontal and left temporal regions, and between the occipital and right temporal regions, may have been affected.

The aforementioned findings reveal a correlation between the energy and complexity indices of EEG and the values of Aβ1‐42/Aβ1‐40. Additionally, in comparison with the negative group, the positive group (Aβ1‐42/Aβ1‐40 < 0.1) exhibited a notable shift in energy within the high‐frequency band, complexity in the frontal area, and mutual information among brain regions. These outcomes imply the viability of utilizing energy and complexity features in EEG as predictive measures for Aβ1‐42/Aβ1‐40 levels.

### 2.2 Microstates analysis in EEG

**FIGURE 2:**
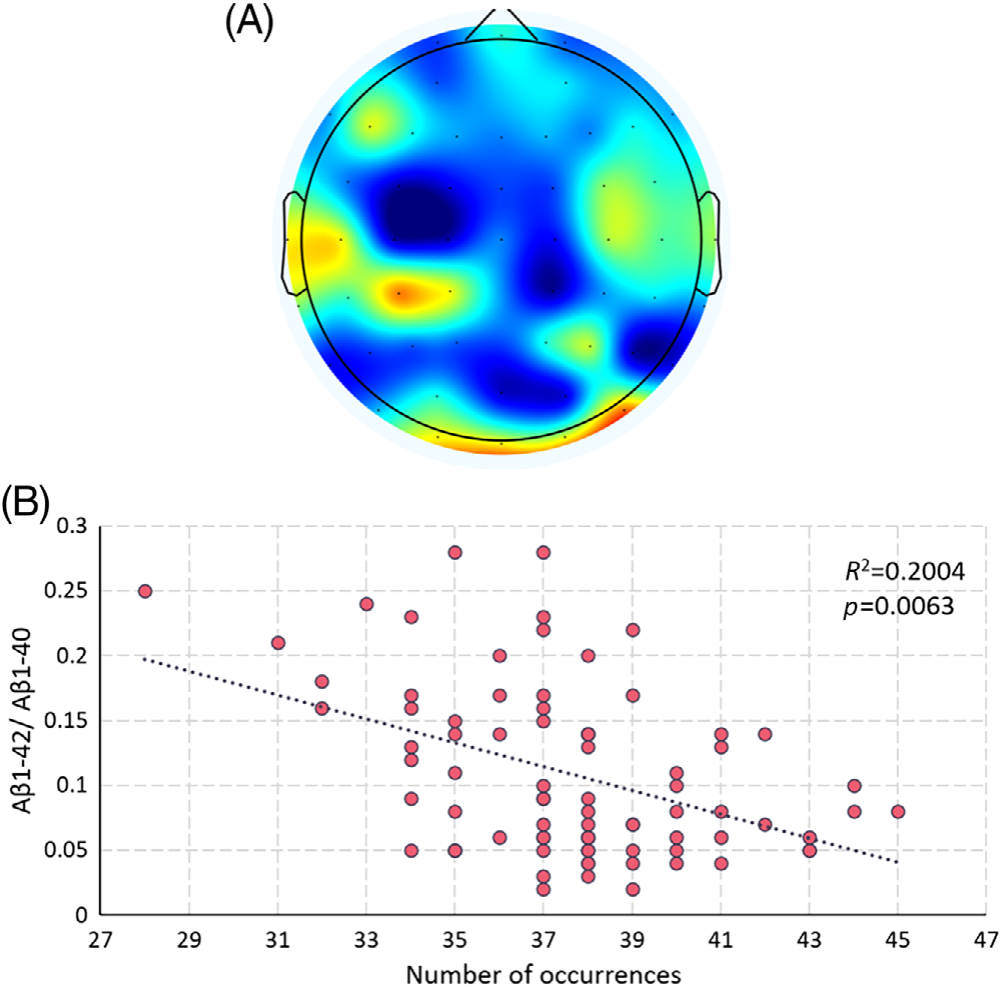
Microstates associated with CSF proteins. The results demonstrate the microstates whose frequency of occurrence significantly correlates with the quantity of CSF makers. The microstate is negatively correlated correlates with value of Aβ1‐42/Aβ1‐40 and the microstate show high activity in the frontal, temporal and occipital lobes. The above summary results include a total of 82 participants (*n* = 82).

The study classified microstates based on the EEG's short‐time stationary properties and experimented with different cluster sizes. When clustered into 7 classes, specific microstates are eventually discovered. The frequency of occurrence of the microstates shown in Figure 2 correlated strongly with the value of Aβ1‐42/Aβ1‐40. This finding implies that the pattern of neural activity depicted in Figure 2 may be influenced by abnormal protein accumulation and may also be related to the protein deposition pathway. Both microstates characterize frontal and temporal lobe activity, which may be related to protein deposition affecting frontal temporal lobe function preferentially. Such a pattern of neural activity may characterize disease progression and have the potential to be a target for disease intervention.

### 2.3 Spatial structure analysis

**FIGURE 3:**
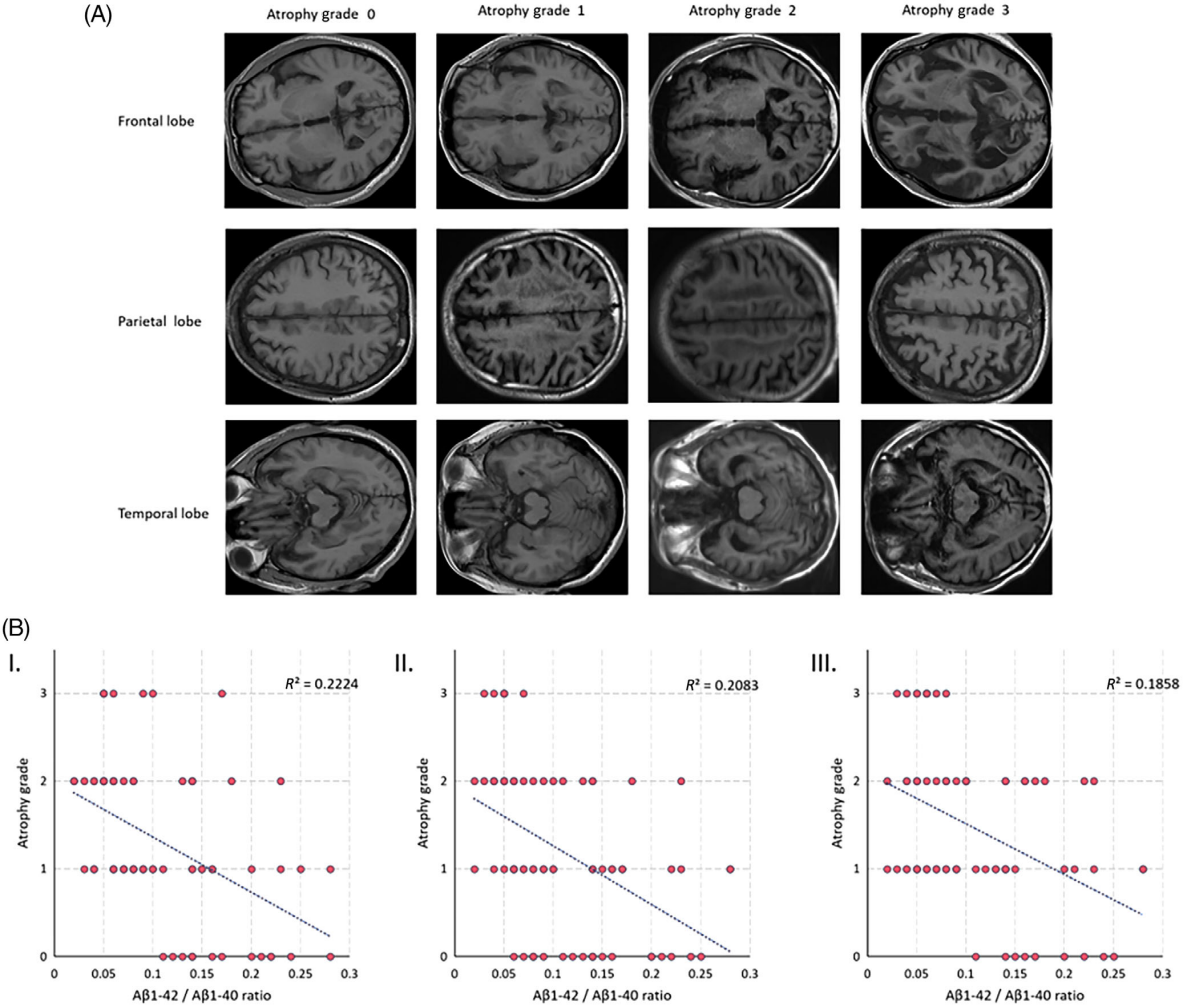
This figure presents the results of spatial feature analysis using MRI. Panel (A) depicts a schematic diagram illustrating the extent of atrophy in the frontal, parietal, and temporal lobes, graded on a scale from 0 to 3 based on the GCA‐F criteria. Accompanying the diagram are representative MRI images of typical subjects. Panel (B) consists of scatter plots I, II, and III, illustrating the distribution of the frontal, parietal, and temporal lobes in relation to changes in the Aβ1‐42/Aβ1‐40 ratio. The black dashed lines depicted in the plots represent the linear fit lines. The above summary results include a total of 77 participants (*n* = 77).

Further, we conducted an analysis to examine the relationship between the values of Aβ1‐42/Aβ1‐40 and alterations in the spatial structure of the brain. Initially, we assessed the extent of atrophy in the frontal, parietal, and temporal lobes using the Global Cerebral Atrophy‐Frontal Sub‐scale (GCA‐F). The degree of atrophy was categorized into four levels (0–3) based on the GCA‐F criteria, as depicted in Figure 3(A), after evaluating the subject's MRI images. Subsequently, we examined the correlation between the degree of atrophy in each brain region and the values of Aβ1‐42/Aβ1‐40, as illustrated in Figure 3(B). The linear fit line clearly demonstrates a gradual reduction in the degree of atrophy across all three brain regions as the Aβ1‐42/Aβ1‐40 value increases. To quantify the association, we calculated Pearson correlation coefficients (*R* ^2^ = 0.22, 0.21, 0.19) between the degree of atrophy in the frontal, parietal, and temporal lobes and the corresponding Aβ1‐42/Aβ1‐40 values. Our findings indicate a moderate correlation. These results substantiate the link between MRI‐derived spatial structural changes in the brain and Aβ1‐42/Aβ1‐40 values, supporting the feasibility of employing MRI structural information to predict Aβ1‐42/Aβ1‐40 values.

### 2.4 Prediction of random forest

The preceding findings indicate correlations between various features of EEG (such as energy, complexity, mutual information, microstates) and MRI (e.g., degree of atrophy) with Aβ1‐42/Aβ1‐40. Hence, these features were employed to predict the values of Aβ1‐42/Aβ1‐40. At the same time, to assess the individual contributions of EEG and MRI to the results, we conducted separate prediction analyses utilizing only the EEG features and the MRI features, as well.

**FIGURE 4:**
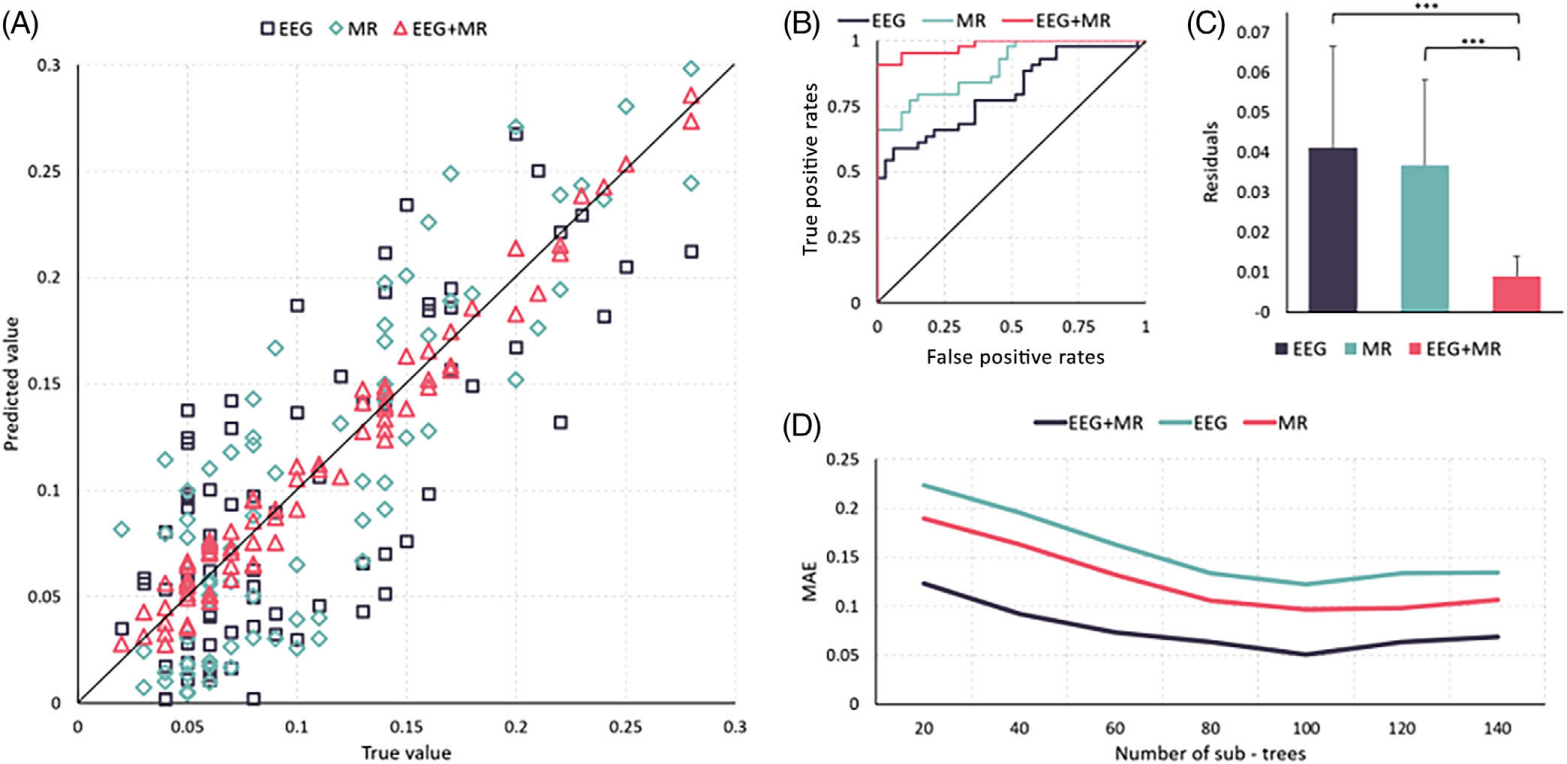
Prediction results of the random forest model. Panel (A) is a scatter plot illustrating the joint distribution of predicted and actual values. The magenta triangle corresponds to data points obtained using both EEG and MRI features, the light green diamond represents results using only MRI features, and the black square denotes results using solely EEG features. Panel (B) shows the ROC curve of predicted results, where the magenta curve represents outcomes with both EEG and MRI features, the light green curve represents results using only MRI features, and the black curve represents results based solely on EEG features. Panel (C) presents the residual plot of the predicted results is displayed. The magenta bars represent results obtained using both EEG and MRI features, the light green bars represent results using only MRI features, and the black bars represent results based solely on EEG features. Notably, there are significant differences observed among all three sets of residuals. Panel (D) presents the trend plot depicting the MAE variation with the number of sub‐trees. The magenta curve corresponds to results utilizing both EEG and MRI features, the light green curve represents outcomes using only MRI features, and the black curve signifies results based solely on EEG features. The above summary results include a total of 77 participants (*n* = 77).

This study combines EEG and MRI features to predict CSF makers. The results show that the prediction model using the random forest method can better predict the Aβ1‐42/Aβ1‐40 ratio, which has high clinical significance for AD diagnosis. The experimental results showed that the mean absolute error was minimized when the number of sub‐decision trees was 100, so 100 decision trees were set up with a minimum of five leaf nodes per tree. The study began with a sevenfold cross‐validation of 82 samples, with a final mean prediction error of 0.0099. Figure 4 shows the mean true and predicted values for each of the 8 samples used as tests. the overall Aβ1‐42/Aβ1‐40 range for the 82 subjects was 0.00002–0.017, and the ratio of the mean prediction error of this method to the total fluctuation range of true values was 0.009 (prediction error: 8.4%). It proves that the present method cloud achieves a more accurate prediction of Aβ1‐42/ Aβ1‐40.

## 3 DISCUSSION

In clinical diagnosis, a critical biomarker for diagnosing AD is the Aβ1‐42/Aβ1‐40 concentration ratio. To compute it, however, requires collecting CSF through painful and costly spinal puncture which is invasive and unpleasant. In this study, noninvasive modalities EEG and MRI were proposed as an alternative to CSF collection. Along with behavioral scale scores, implications from these two modalities were expected to serve as a potential AD diagnosis criterion.

To have a higher interpretability in clinical diagnostic settings, we first investigated the relativity of EEG and MRI with the clinical‐recognized Aβ1‐42/Aβ1‐40 values. CSF tests, MRIs, and EEGs of 63 patients with mild cognitive impairment (MCI) and dementia were collected. Strong correlation was observed between multiple features in EEG and MRI with the biochemical parameter, including EEG frequency feature, signal complexity, signal coupling, and MRI structural features. We then employed machine learning approaches and developed a system of CSF amyloid beta protein prediction based on multimodality data of behavioral scale, EEG and MRI data of MCI and dementia patients. This system showed a great performance with the mean error of the prediction to be less than 0.01. This finding built a solid and reliable bridge between new, noninvasive data modality and traditional, invasive biochemical biomarkers. As a result, this would add to the expanding body of literature on noninvasive approaches for evaluating biomarkers linked with AD and diagnostic applications.

The use of techniques in this study offers several advantages, including their noninvasive nature, cost effectiveness, and wide availability in clinical settings. This study highlights their utility in providing a complementary approach to traditional invasive methods for biomarker assessment. The ability to noninvasively estimate Aβ levels in the CSF has significant implications for early AD detection and monitoring disease progression. By providing an accessible and efficient means of assessing Aβ burden, EEG and MR techniques may enhance clinical decision‐making and facilitate personalized treatment strategies.

Furthermore, our study adds to the existing evidence regarding the association between Aβ pathology and aberrant brain activity. The observed relationships between EEG/MR measures and Aβ levels lend support to the hypothesis that AD‐related changes in Aβ deposition may have a direct impact on neural functioning.^30^ Future research should aim to elucidate the underlying mechanisms linking Aβ pathology, EEG/MR abnormalities, and cognitive decline in AD.

This study's limitations are important to consider. Firstly, the relatively small sample size may limit the generalizability of our findings, potentially impacting the robustness and statistical power of our analyses. Additionally, our study's cross‐sectional design hinders the ability to track changes in Aβ levels over time, suggesting that future investigations should include longitudinal approaches using EEG and MR measures. Furthermore, the specific EEG and MR protocols employed in this study need careful evaluation and standardization for consistent and reproducible results in various research settings. It is also crucial to recognize that our findings may not be applicable to all demographic groups, given the particular characteristics of our study population. Expanding future studies to include a more diverse cohort would enhance the applicability of the results and provide a more comprehensive understanding of AD progression and diagnosis. Given the variability of the scalp EEG signaling pathway, methods to increase the scalp EEG signal‐to‐noise ratio such as Minimally Invasive Local‐Skull Electrophysiological Modification^31^, ^32^ or Neural Dust^33^, ^34^, ^35^ might also help to further improve this study. More accurate and faster MRI imaging may also help the results.^36^ In the study, participants included 45 females and 37 males, resulting in a gender ratio of 1.2. This gender distribution may also have a minimal impact on the results of the research.

In conclusion, this study highlights the potential of EEG and MR techniques as noninvasive predictors of Aβ1‐42/Aβ1‐40 levels in the CSF. The ability to estimate Aβ burden through these neuroimaging methods opens up new possibilities for early AD detection, monitoring disease progression, and informing personalized treatment approaches. Future research should continue to explore the utility of these techniques in larger cohorts, longitudinally, and in combination with other biomarkers to enhance our understanding of AD pathophysiology and improve clinical outcomes.

## 4 MATERIALS AND METHODS

### 4.1 Participants and ethics

The study included patients with cognitive decline complaints from the Memory Specialist Clinic of the Department of Neurology at Tongji Hospital, Tongji University. Ethical approval was granted by the Ethics Committee of Tongji Hospital, Shanghai, China. All participants provided informed consent. Patients were eligible for the study if they met the following criteria: (1) age over 50 years with no gender restriction; (2) absence of brain tumor, epilepsy, neurosyphilis, new cerebral infarction at the time of consultation, or other central nervous system diseases (infections, clear history of demyelinating diseases, etc.); (3) no significant physical medical conditions, such as hepatic encephalopathy or myocardial infarction; (4) no previous history of serious mental disorders, psychoactive substance or drug abuse; (5) able to cooperate with the examination, complete the full neuropsychological assessment, and sign an informed consent form; and (6) no contraindications to cranial MRI or EEG. A total of 82 patients were enrolled.

**FIGURE 5:**
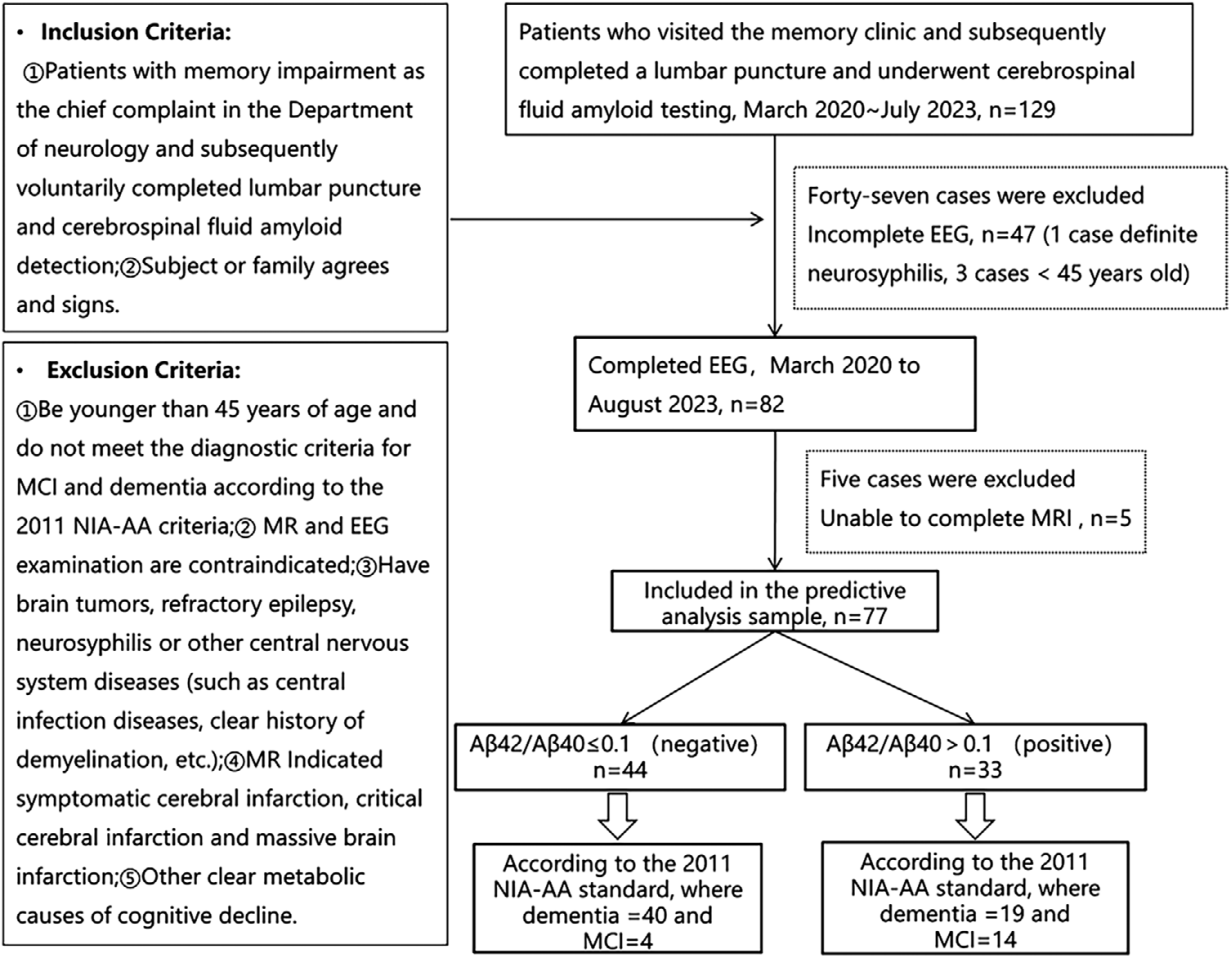
Flow chart of inclusion/exclusion criteria. In the initial cohort according to the standard criteria, 129 participants were enrolled, out of which 82 completed EEG recordings. Among these, 77 participants also completed MR recordings. The study ultimately utilized data from these 77 participants for predicting CSF biomarkers.

Based on the inclusion and exclusion criteria shown in Figure 5, a total of 82 participants completed EEG data collection. They were grouped according to their Aβ42/Aβ40 ratio. The positive group (Aβ42/Aβ40 ≤ 0.1) included 47 individuals with an average age of 71.62 ± 9.85, while the negative group (Aβ42/Aβ40 > 0.1) comprised 35 individuals with an average age of 69.34 ± 7.72. Cognitive assessments were conducted for both groups according to the 2011 NIA‐AA diagnostic criteria. The results are as follows: In the Aβ42/Aβ40 > 0.1 group, there were 15 participants with MCI and 20 participants with dementia. In the Aβ42/Aβ40 ≤ 0.1 group, there were 4 MCI participants and 43 dementia participants. A total of 77 participants completed both EEG and MRI scans. In the positive group (Aβ42/Aβ40 ≤ 0.1), there were 44 individuals with an average age of 71.45 ± 9.91, including four MCI and 40 dementia participants. In the negative group (Aβ42/Aβ40 > 0.1), there were 33 individuals with an average age of 69.24 ± 7.84, including 14 MCI and 19 dementia participants.

### 4.2 EEG data acquisition and preprocessing

EEG data in this study were collected using a 64‐lead EEG recording system from Brain Products following the International 10−20 EEG Society standards for electrode placement. The time constant was set to 0.3 s, and band‐pass filtering was applied within the range of 0.5–100 Hz. The electrode contact resistance was less than 5 kΩ. Prior to task EEG recording, all subjects underwent at least 5 min of resting EEG with their eyes closed. EEG signal preprocessing included electrode positioning, band‐pass filtering, rejection of noninformative channels, threshold‐based rejection of bad segments, independent component analysis to remove electromyographic artifacts, and whole‐brain average re‐referencing. MATLAB 2018b platform was used for EEG signal processing in this research.

### 4.3 CSF collection and testing

The CSF was obtained through lumbar puncture, performed between the L3/L4 or L4/L5 intervertebral spaces while the patient was in the lateral position. The CSF was collected in 5 mL polypropylene tubes using a standard needle. The collected samples were then centrifuged at 1568 g for 10 min, divided into 2 mL polypropylene tubes, and stored at −80°C until analysis. Analysis was conducted by EUROIMMUN Medical Laboratory Diagnostics Stock Company, which used four kits (Total‐Tau, p‐Tau(181), Beta‐Amyloid(1‐40), Beta‐Amyloid(1‐42)) for measurement of Aβ42, Aβ40, p‐tau, and t‐tau concentrations via enzyme‐linked immunosorbent assay. The study categorized participants into negative and positive groups based on an Aβ1‐42/Aβ‐40 threshold of 0.1.^37^ Statistical analysis was conducted to identify differences in EEG and MRI features between these groups. Subsequently, these characteristics were used to develop a predictive model for this biomarker.

### 4.4 MRI data acquisition and evaluation

MRI was performed using a 3.0T MRI system (Magneton Verio; Siemens Medical Systems, Erlangen, Germany) with a standard orthogonal head coil prior to acquisition. During the MRI scan, the patient was sedated and the head was held in place by a sponge. Conventional sagittal T1, axial T1, T2, T2*, T2 fluid‐attenuated inversion recovery sequence images and diffusion‐weighted imaging were acquired. A total of 21 slices were obtained using the AC‐PC line as the baseline.

### 4.5 Brain atrophy grade assessment (GCA‐F)

The GCA‐F is defined in the frontal cortex and sulcal. It ranks 0–3, with grade 0 representing no cortical atrophy; grade 1, mild atrophy (dilatation of sulci); grade 2, moderate atrophy (loss of gyri volume); and grade 3, end‐stage “knife blade atrophy.”^38^

### 4.6 Linear measurement of brain atrophy based on MRI

The following linear measurements were measured in the sagittal and axial directions of T1 by using digital calipers: maximal transversal intracranial width (A), maximal longitudinal intracranial width (B), maximal frontal horn width (E), minimal inter‐caudate distance (F), choroid plexuses distance (J), maximal width of third ventricle (H), temporal horn width (O), suprasellar cistern width (P), corpus callosum genu (a), corpus callosum body (b), corpus callosum sub (c). The following features were calculated from the measurements: Evans Ratio, Bicaudate Ratio, Huckman Number, Huckman Ratio, Third Ventricular Ratio, Ventricle Index, Temporal Horn Ratio, and Suprasellar Cistern Ratio.^39^, ^40^ The precise anatomical locations for these measurements are depicted in Figure S1, while the definitions and computational methodologies for the aforementioned indices and ratios are detailed in Tables S2 and S3.

### 4.7 EEG data processing

#### 4.7.1 Frequency feature computation

The power distribution of the signal at different frequencies can be obtained using the fast Fourier transform (FFT). The input signal is a superimposed average of 20 consecutive segments containing 2000 sampling points. Results of the power distribution in the frequency domain were obtained with a frequency domain resolution of 0.5 Hz. The point with the highest power in the alpha band is selected as the dominant frequency in the band.

#### 4.7.2 L–Z signal complexity

This study also used L–Z signal complexity^41^ to evaluate the probability of emerging signal patterns and the feature of neural activity transitions.

(1) $$S_{n}=\left\{\begin{array}{c} 0\;x_{n}\leq T_{1}\\ 1\;T_{1}<x_{n}\leq T_{2}\\ \;\;\vdots \\ l\;T_{l}<x_{n} \end{array}\right.,\;\;C\;=\frac{c\mathrm{lo}g_{l}n}{n}\;$$

In the formula, the original signal (formula1) is segmented into a new sequence S according to l segments. And then the normalized complexity of the S sequence is calculated,^41^ in which c is the complexity of sequence S.

#### 4.7.3 Signal coupling computation

The activity of the nervous system is not usually the result of arousal and silence in a single brain region. Its information processing is often accompanied by the collaboration of multiple brain regions. In order to evaluate the information interaction of neural activity at different locations, the present work calculates the mutual information of signals from different leads to characterize the information coupling capacity between several brain regions. The mutual information is a measure of the amount of information contained in each other between two variables and is calculated as follows.

(2) $$I\;\left(X,Y\right)=\sum_{x\in X}\sum_{y\in Y}\left(x,y\right)\log\left(\frac{p\left(x,y\right)}{p\left(x\right)p\left(y\right)}\right)\;$$

Both *X* and *Y* in the formula are EEG signals, while *p*(*x*) and *p*(*y*) are the distributions of *X* and *Y*, respectively. And *p*(*x*,*y*) is the joint distribution of *X* and *Y*. *I* is the mutual information of *X* and *Y*. In practical calculations, the histogram can be adapted to estimate the distribution of the signal.

#### 4.7.4 EEG microstates

EEG is a short‐time stationary nonlinear electrical signal, which means that the overall pattern of neural activity in the EEG does not change significantly over a 50−120 ms time window. In this study, microstates were classified according to a 100 ms time window, and the standard deviation of each lead EEG over 100 ms was calculated as the intensity of activity in that lead over that period of time. Then the microstate category determination using a k‐means method. This study attempted more microstate categories (5–20) than most reports to improve the spatial resolution of the microstates in order to find states that were more relevant to the CSF maker.

### 4.8 Random forest

Random Forest is a commonly used ensemble learning method.^42^, ^43^ As this study combines EEG and MRI multi‐parameter feature information, the random forest method is utilized for combined regression prediction. Random forest consists of a combination of multiple decision trees. The number of decision trees used in this study is determined based on the results of several experiments, and it is set to a value where the number of leaves of the decision tree is five.

To prepare for the regression analysis, the features listed in Table S2 are first correlated with the values to be predicted (Aβ1‐42/Aβ‐40), and then ranked according to the absolute value of their correlation coefficients from largest to smallest. The top 500 features from the 7363 ranked features are selected to obtain the best prediction results. A total of 63 subjects with common EEG and structural information are collected for the training model. Out of these 63 subjects, 50 are used for training, and 8 are used for testing in an eightfold cross‐validation process. In the last validation trial, 55 subjects are used for training, and 8 subjects are used for testing. There is no overlap between the training and test sets. The final result of the model is taken as the average of the results obtained from the entire process, which shows in Table S4.

### 4.9 Statistical and correlation analysis

In the statistical analysis of this study, we employed the two‐sample t‐test to assess the significance of differences between the groups. This choice of test was based on its appropriateness for comparing the means of two independent samples. For this research, a p‐value threshold of 0.05 was set to determine statistical significance. This means that differences with a *p* value less than 0.05 were considered statistically significant. The *p* value is a measure of the probability that the observed difference or a more extreme one would occur by chance if the null hypothesis (no true difference between groups) were true. A *p* value less than 0.05 thus indicates a less than 5% chance that the observed differences are due to random variation alone, suggesting that the observed differences are likely to reflect true differences between the groups. In the correlation analysis section of the study, research utilized the Pearson correlation coefficient to analyze the relationship between two sets of features. The result of this analysis is a number ranging from −1 to 1. A value closer to 0 indicates no correlation between the two sets of features, while a value approaching 1 suggests a positive correlation. Conversely, a value near −1 indicates a negative correlation between the feature sets. This method allows for a quantitative assessment of the degree and direction of association between the variables under study. Due to prioritizing the magnitude of the correlation, the square of the correlation coefficient, *R*, is often calculated. This squared value, known as *R* ^2^, is used to assess the strength and significance of the relationship between variables in statistical analysis.

## AUTHOR CONTRIBUTIONS

J. S. and Y. S. wrote this article. Y. L. and B. L. provided idea as well as financial support for this study. A. S. revised this article. Z. X., R. L., and X. Y. collected the data. All authors have read and approved the final manuscript.

## CONFLICT OF INTEREST STATEMENT

The authors declare no conflict of interest.

## ETHICS STATEMENT

The study was approved by the Ethics Committee of Shanghai Tongji Hospital [(Tong) Audit No. (2021‐LCYJ‐002‐1)], and all subjects signed an informed consent form.

## Supporting information

Supporting information

## Data Availability

The data that support this study are available from the corresponding author upon reasonable request.
