# Supplementary material for: Precise prediction of cerebrospinal fluid amyloid beta protein for early Alzheimer's disease detection using multimodal data
Source: MedComm (2020). 2024 Apr 19;5(5):e532. doi: 10.1002/mco2.532 (PMC11027992; doi:10.1002/mco2.532)
Supplement: Supplementary file 1 — Supporting information [file MCO2-5-e532-s001.docx]

#

# Supporting Information for

# Precise Prediction of Cerebrospinal Fluid Amyloid Beta Protein for Early Alzheimer's Disease Detection Using Multimodal Data

**Jingnan Sun^1, +^, Zengmai Xie^2,3, +^, Yike Sun^1, +^, Anruo Shen**^1^**, Renren Li^2,3^, Xiao Yuan^2,3^, Bai Lu**^5,6, *^**, Yunxia Li** ^2,3,4,^ **^*^**

^+^J.S., Y. S. and Z.X. contributed equally to this work.

^*^To whom correspondence may be addressed. Email: Doctorliyunxia@163.com; bai_lu@tsinghua.edu.cn.

^1^Department of Biomedical Engineering, Tsinghua University, Beijing, 100084, China.

^2^Department of Neurology , Shanghai Pudong Hospital, Fudan University Pudong Medical Center, Shanghai 201399, China

^3^Shanghai Key Laboratory of Vascular Lesions Regulation and Remodeling

^4^Department of Neurology, Tongji Hospital，School of Medicine, Tongji University, Shanghai 200092,China.

^5^School of Pharmaceutical Sciences, Tsinghua University, Beijing, 100084, China.

^6^ Beijing Academy of Artificial Intelligence, Beijing, 100080, China

**Table S1.** Participants basic clinical information

| **Characteristics** | | **Overall**  **(N=82)** |
| --- | --- | --- |
| Age (mean (SD)), years | | 70.57 (8.69) |
| Gender (%) | F | 45 (54.9) |
|  | M | 37 (45.1) |
| BMI (mean (SD)) | | 23.15 (3.67) |
| Education Years (median [IQR]) | | 12.00 [9.00, 15.00] |
| Education Level (%) | MissingData | 3 ( 3.7) |
|  | High school diploma or above | 45 (54.9) |
|  | Primary | 11 (13.4) |
|  | Secondary | 23 (28.0) |
| Tobacco (%) | MissingData | 2 ( 2.4) |
|  | Current | 5 ( 6.1) |
|  | Former | 17 (20.7) |
|  | Never | 58 (70.7) |
| Alcohol (%) | MissingData | 2 ( 2.4) |
|  | Current | 4 ( 4.9) |
|  | Former | 4 ( 4.9) |
|  | Never | 72 (87.8) |
| Stroke (%) | MissingData | 2 ( 2.4) |
|  | No | 66 (80.5) |
|  | Yes | 14 (17.1) |
| Hypertension (%) | MissingData | 2 ( 2.4) |
|  | No | 47 (57.3) |
|  | Yes | 33 (40.2) |
| Diabetes (%) | MissingData | 2 ( 2.4) |
|  | No | 66 (80.5) |
|  | Yes | 14 (17.1) |
| Dyslipidemia (%) | MissingData | 2 ( 2.4) |
|  | No | 61 (74.4) |
|  | Yes | 19 (23.2) |
| APOE (%) | MissingData | 29 (35.4) |
|  | E2/E3 | 6 ( 7.3) |
|  | E2/E4 | 2 ( 2.4) |
|  | E3/E3 | 22 (26.8) |
|  | E3/E4 | 19 (23.2) |
|  | E4/E4 | 4 ( 4.9) |
| MMSE (median [IQR]) | | 20.00 [14.00, 23.50] |
| ADL (median [IQR]) | | 21.00 [17.00, 26.00] |
| NPI (median [IQR]) | | 2.00 [1.00, 10.00] |
| MTA_R (%) | 0 | 4 ( 5.2) |
|  | 1 | 14 (18.2) |
|  | 2 | 20 (26.0) |
|  | 3 | 17 (22.1) |
|  | 4 | 22 (28.6) |
| MTA_L (%) | 0 | 6 ( 7.8) |
|  | 1 | 12 (15.6) |
|  | 2 | 20 (26.0) |
|  | 3 | 17 (22.1) |
|  | 4 | 22 (28.6) |
| PWMH (%) | 0 | 1 ( 1.3) |
|  | 1 | 27 (35.1) |
|  | 2 | 20 (26.0) |
|  | 3 | 29 (37.7) |
| DWMH (%) | 0 | 33 (42.9) |
|  | 1 | 13 (16.9) |
|  | 2 | 9 (11.7) |
|  | 3 | 22 (28.6) |
| CMBs (%) | MissingData | 5 ( 6.1) |
|  | No | 70 (85.4) |
|  | Yes | 7 ( 8.5) |
| Lacunes (%) | MissingData | 5 ( 6.1) |
|  | No | 47 (57.3) |
|  | Yes | 30 (36.6) |


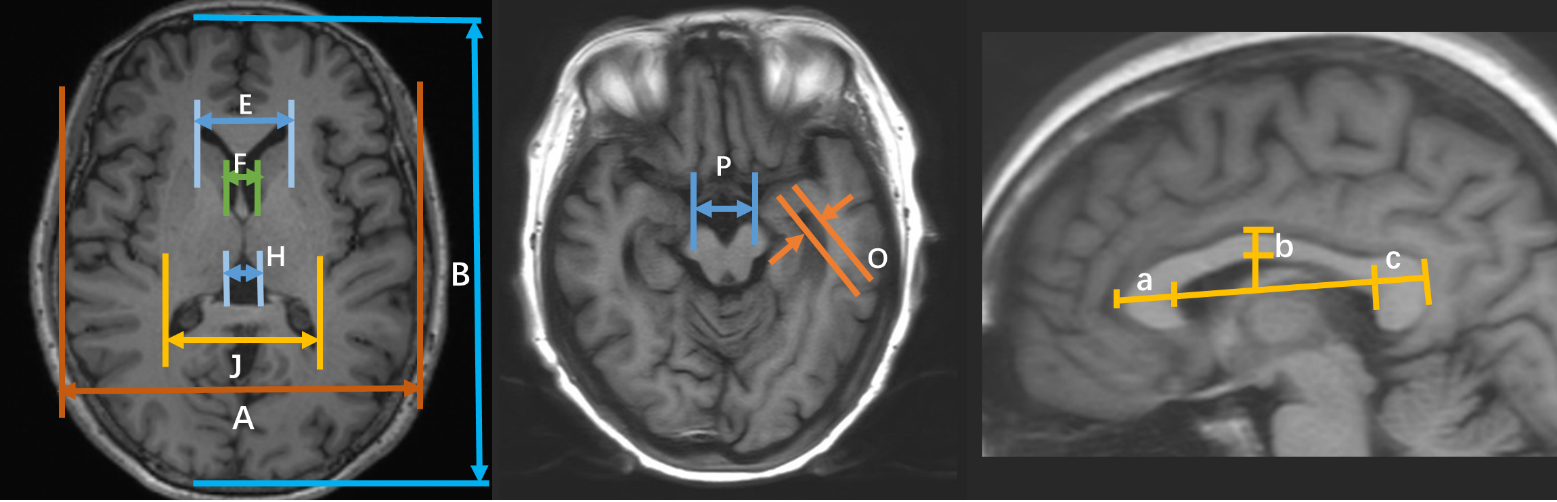


**Figure S1.** Linear measurements taken on sagittal and axial MRI slices. Maximal transversal intracranial width (A), Maximal Longitudinal Intracranial Width (B), Maximal Frontal Horn Width (E), Minimal Inter-caudate Distance (F), Choroid Plexuses Distance (J), Maximal Width of Third Ventricle (H), Temporal Horn Width (O), Suprasellar Cistern Width (P), Corpus callosum genu (a), Corpus callosum body (b), Corpus callosum sub (c). the images were oriented by the interhemispheric fissure, the vein of Galen, and the cerebral aqueduct from T1-weighted sequences (in each patient the same sequence was used).

**Table S2.** MRI Measured features

| **Measured features** | **mark** |
| --- | --- |
| Maximal Transversal Intracranial Width (MTIW) | A |
| Maximal Longitudinal Intracranial Width (MLIW) | B |
| Maximal Frontal Horn Width (MFHW) | E |
| Minimal Inter-caudate Distance (MID) | F |
| Choroid Plexuses Distance (CPD) | J |
| Maximal Width of Third Ventricle (MWTV) | H |
| Temporal Horn Width (THW) | O |
| Suprasellar Cistern Width (SCW) | P |
| Corpus Callosum Genu | a |
| Corpus Callosum Body | b |
| Corpus Callosum Sub | C |

**Table S3.** MRI Calculated features

| **Calculated features** | **Calculation method** |
| --- | --- |
| Evans Ratio | E/A |
| Bicaudate Ratio | F/A |
| Huckman Number | E+F |
| Huckman Ratio | (E+F)/A |
| Third Ventricular Ratio | H/A |
| Ventricle Index | J/E |
| Temporal Horn Ratio | O/A |
| Suprasellar Cistern Ratio | P/A |

**Table S4.** Features for random forest prediction

| **Feature** | **Computation** | **Number of inputs** |
| --- | --- | --- |
| frequency feature | Fast Fourier transform | 54*81（lead*frequency） |
| signal complexity | Lem-Ziv Complexity | 54（lead） |
| signal coupling | mutual information | 54*54（lead * lead） |
| structural features | standardized statistics | 11+8 (measured +computed） |
| behavioral scales | MMSE | 1 (score) |
